# Molecular Level Structure of Biodegradable Poly(Delta-Valerolactone) Obtained in the Presence of Boric Acid

**DOI:** 10.3390/molecules23082034

**Published:** 2018-08-14

**Authors:** Khadar Duale, Magdalena Zięba, Paweł Chaber, Dany Jeanne Di Fouque, Antony Memboeuf, Cristian Peptu, Iza Radecka, Marek Kowalczuk, Grażyna Adamus

**Affiliations:** 1 Centre of Polymer and Carbon Materials, Polish Academy of Sciences, 34, M. Curie-Sklodowska St., 41-819 Zabrze, Poland; mzieba@cmpw-pan.edu.pl (M.Z.); pchaber@cmpw-pan.edu.pl (P.C.); marek.kowalczuk@cmpw-pan.edu.pl or M.Kowalczuk@wlv.ac.uk (M.K.); 2 CEMCA, Université de Bretagne Occidentale, 6 Av. Le Gorgeu, 29238 Brest CEDEX 3, France; fouque@univ-brest.fr (D.J.D.F.); memboeuf@univ-brest.fr (A.M.); 3 Polymer Institute, Slovak Academy of Sciences, Dúbravská cesta 9, 845-41 Bratislava 45, Slovakia; upolcris@savba.sk or cristian.peptu@icmpp.ro; 4 “Petru Poni” Institute of Macromolecular Chemistry, Alee Grigore Gica Voda 41A, 700487 Iasi, Romania; 5 School of Biology, Chemistry and Forensic Science, Faculty of Science and Engineering, University of Wolverhampton, Wolverhampton WV1 1SB, UK; I.Radecka@wlv.ac.uk (I.R.)

**Keywords:** Poly(delta-valerolactone), delta-valerolactone, spectrometry, biodegradable polymers, metal free catalyst, MALDI, ESI-MS, ring-openings polymerizations (ROP), biocatalyst, biodegradable aliphatic polyesters, MS/MS studies

## Abstract

In this study, low molecular weight poly(*δ*-valerolactone) (PVL) was synthesized through bulk-ring openings polymerization of *δ*-valerolactone with boric acid (B(OH)_3_) as a catalyst and benzyl alcohol (BnOH) as an initiator. The resulting homopolymer was characterized with the aid of nuclear magnetic resonance (NMR) and mass spectrometry (MS) techniques to gain further understanding of its molecular structure. The electrospray ionization mass spectrometry (ESI-MS) spectra of poly(*δ*-valerolactone) showed the presence of two types of homopolyester chains—one terminated by benzyl ester and hydroxyl end groups and one with carboxyl and hydroxyl end groups. Additionally, a small amount of cyclic PVL oligomers was identified. To confirm the structure of PVL oligomers obtained, fragmentation of sodium adducts of individual polyester molecules terminated by various end groups was explored in ESI-MS^n^ by using collision induced dissociation (CID) techniques. The ESI-MS^n^ analyses were conducted both in positive- and negative ion mode. The comparison of the fragmentation spectra obtained with proposed respective theoretical fragmentation pathways allowed the structure of the obtained oligomers to be established at the molecular level. Additionally, using matrix-assisted laser desorption ionization time-of-flight mass spectrometry (MALDI-TOF-MS), it was proven that regardless of the degree of oligomerization, the resulting PVL samples were a mixture of two types of linear PVL oligomers differing in end groups and containing just a small amount of cyclic oligomers that tended to be not visible at higher molar masses.

## 1. Introduction

Lactones are cyclic esters and a very important polymer building material. The synthetic aliphatic polyesters derived from lactones have a unique position among biodegradable polymers. They have been found to have various health-related applications due to their good biodegradability and biocompatibility properties [1,2]. Many practical applications of these materials in medicine arise from their degradability in vivo, which can be achieved through simple hydrolysis of the ester backbone in aqueous environments, such as body fluids, to form products that are ultimately broken down to nontoxic metabolites [3,4,5]. Therefore, this group of polymers has been widely investigated both in terms of synthesis pathway as well as in structural characterizations [6,7,8,9,10,11].

Among the available synthetic aliphatic polyesters, attention for medical applications has mostly focused on polylactide (PLA), poly(3-hyroxybutyrate) (PHB), and poly(ε-caprolactone) (PCL) as well as their copolymers [12,13,14]. Much less attention has been devoted to poly(*δ*-valerolactone) (PVL) to date. PVL is an established biopolymer and has found several important applications in drug formulation and delivery systems [15,16,17,18]. PVL-based polymers have also been used as carriers to improve drug skin delivery of certain antifungal medicines [19] and as a hydrophobic block in amphiphilic block copolymers for in vivo drug delivery systems of chemotherapy drugs, such as the anthracycline antibiotic of daunorubicin (DNR), doxorubicin (DOX), and many other hydrophobic drugs [20,21]. Anthracycline antibiotics are large spectrum antitumor drugs used in the treatment of most cancer types, including various lung, breast, bladder, ovarian, brain cancers, Kaposi’s sarcoma, lymphoma, and acute lymphocytic leukemia [22].

A lot of work has been dedicated to structural characterization of biodegradable and biocompatible aliphatic polymers, which is crucial for the wider application of polymers. Structural characterization of synthetic biodegradable polymers is essential to determine the compatibility of their application in many different fields [10,23,24,25]. These include medicine, cosmetology, groundwater chemistry, and bioreactors. In particular, polymer materials that may be suitable for medicinal applications require precise characterization in order to recognize the relationship between structure and properties. 

It is well documented that considerable advances have been made in recent years regarding polymer mass spectrometry analysis; both matrix-assisted laser desorption ionization time-of-flight mass spectrometry (MALDI­TOF­MS) and electrospray ionization mass spectrometry (ESI-MS) have played a vital role in this field [23,24,25,26,27,28,29]. The use of ESI-MS and MALDI­TOF­MS has accelerated precise structural characterization, including end group analysis. MALDI­TOF­MS and ESI­MS are suitable for polymer analysis because they are sensitive and nonaveraging techniques that provide detailed information about the structure of individual molecules (including end group analysis) in a polymer sample [27,28,30,31]. These techniques are also widely used with increasing success for characterization of synthetic copolymers. Furthermore, a deeper insight into the structure of homopolyesters and copolyesters can be achieved by means of mass spectrometric fragmentation techniques. The principal advantage of using this technique is that more information is available than what can be obtained by direct analysis using conventional (single stage) mass spectral methods [31,32,33].

In this paper, we report the synthesis of biodegradable poly(*δ*-valerolactone) via a bulk-ring opening polymerization of *δ*-valerolactone with the use of boric acid as a nontoxic metal-free catalyst. Taking into consideration the prospective use of the synthesized polyester in the medical field, it was important to elucidate the structure and homogeneity of the obtained polymeric products.

Herein, we also focus on the application of the electrospray multistage mass spectrometry (ESI­MS^n^) and MALDI­TOF­MS for structural characterization at the molecular level of the potential biomaterials. The structure of individual macromolecules of the resulting poly(*δ*-valerolactone) (including the chemical structure of their end groups) was determined using ESI-MS^n^ supported by proton nuclear magnetic resonance (^1^H-NMR) spectroscopy. The chemical structure of the individual poly(*δ*-valerolactone) chains was unequivocally established by investigating the fragmentation product patterns of the individual molecular ions.

## 2. Results and Discussion

In recent years, much attention has been focused on the use of lactones for the synthesis of new biodegradable homo- and copolymers with specific characteristics, e.g., for prospective applications in the medical field [16]. The knowledge of structure-property relationships is essential for the successful application of polymeric materials, especially in biomedical applications. Therefore, there is a continuous requirement for the development and detailed characterization of polymeric materials at the molecular level.

**Scheme 1:**
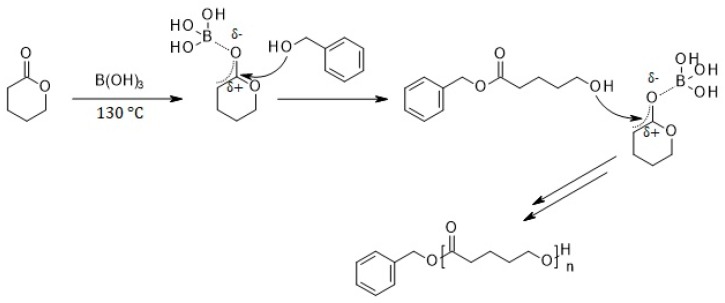
The ring-opening polymerization of *δ*-valerolactone initiated by benzyl alcohol with the presence of boric acid as a catalyst to activate the monomer.

The main purpose of the research described in this paper was to show the usefulness of mass spectrometry techniques with soft ionization method in the molecular level structure studies of new biodegradable aliphatic polyesters. The object of the present study—poly(*δ*-valerolactone)—was synthesized based on previous studies that have demonstrated the feasibility of boric acid in catalyzing the ring-openings polymerization of higher lactones [7]. Scheme 1 describes the ring-opening polymerization of *δ*-valerolactone (*δ*-VL) initiated by benzyl alcohol with the presence of boric acid as a catalyst. The obtained poly(*δ*-valerolactone) polyesters were preliminarily characterized by gel permeation chromatography (GPC), ^1^H-NMR, and particularly by ESI tandem mass spectrometry (ESI-MS/MS) and MALDI-TOF-MS/MS techniques. The results of characterization of the polymers obtained by GPC are summarized in Table 1. 

### 2.1. The ^1^H-NMR Studies

**Figure 1:**
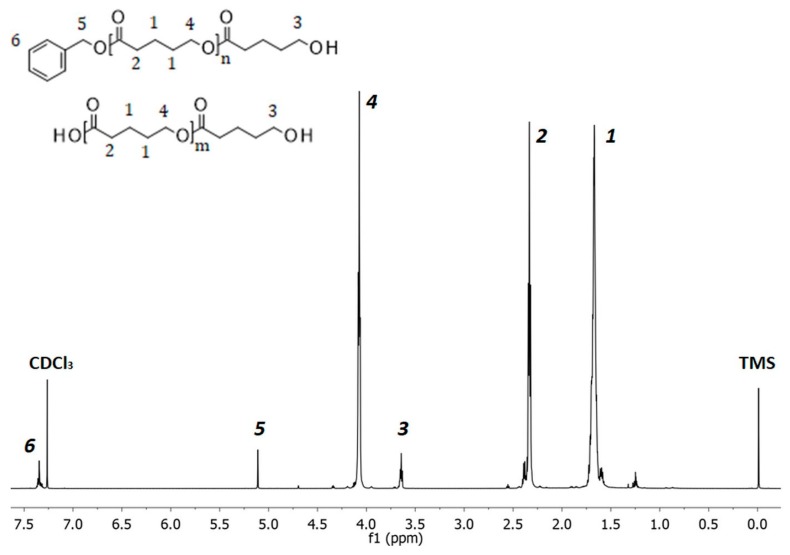
Proton nuclear magnetic resonance (^1^H-NMR) spectrum of poly(*δ*-valerolactone) oligomers (Sample 1, Table 1).

The general chemical structure of obtained polyester in this study was determined by ^1^H-NMR spectroscopy and is presented in Figure 1. The ^1^H-NMR spectrum reveals signals ascribed to the protons of the methylene groups of valerate repeating units (corresponding signals **1, 2** and **4**). Additionally, the signals corresponding to the protons of benzyl moiety (**5** and **6**) (derived from the incorporated into some polymeric chains initiator) and protons of methylene groups (**3**) connected with hydroxyl terminal groups were detected in the spectrum.

### 2.2. ESI-MS Analysis

**Figure 2:**
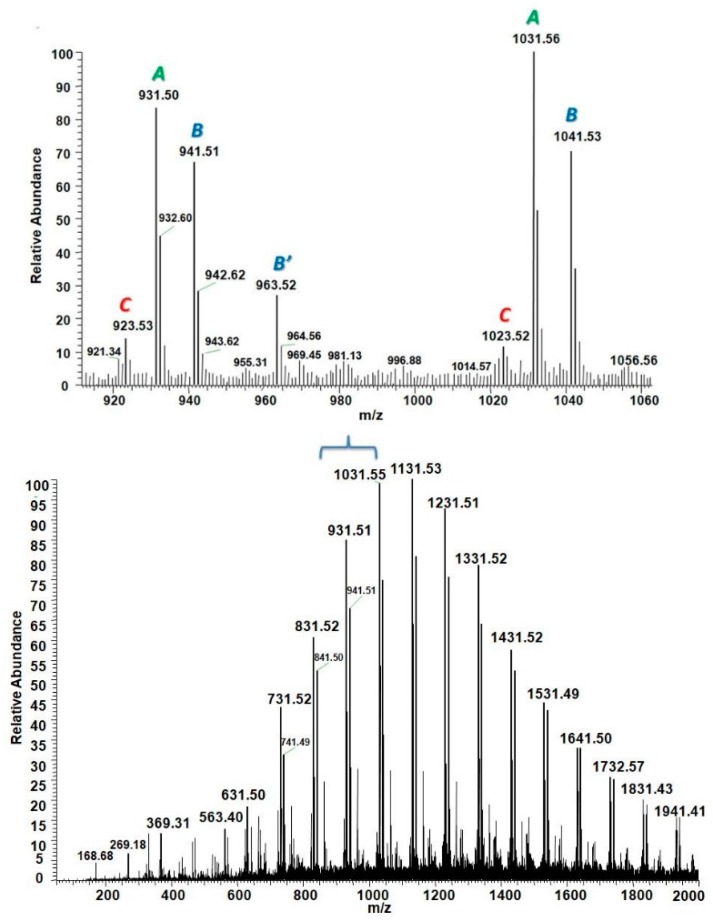
ESI-MS spectrum (positive-ion mode) of PVL oligomers and the spectral expansion in the range *m/z* 910–1050 (Sample 1, Table 1).

**Figure 3:**
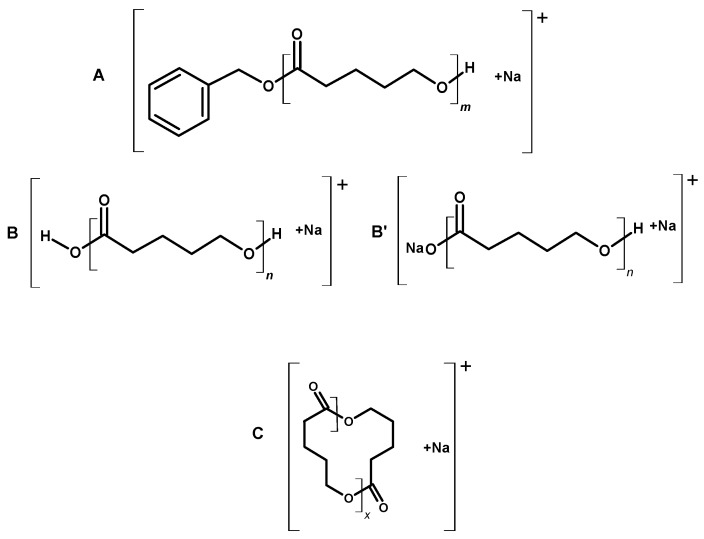
The structures of the ions observed in ESI-MS of PVL.

To obtain detailed information about the structure at the molecular level of the resulting poly(*δ*-valerolactone), ESI-MS was used. The ESI-mass spectrum (positive ion mode) of poly(*δ*-valerolactone), together with spectral expansion in the mass range *m*/*z* 910–1050, are depicted in Figure 2. The observed ions may be structurally assigned based on their m/z value. Four series of singly charged ions with a peak-to-peak mass increment of 100 Da that correspond to three types of oligomers present in the mass spectrum are shown in Figure 3. The main series (labeled as A series) of ions that appears in the mass spectrum at *m/z* = 107 + (n × 100) + 1 + 23—where 107 is the molar mass of C_6_H_5_CH_2_O end group, 100 and n are molar mass and number of *δ*-valerolactone repeating units, respectively, 1 is the mass of proton and 23 is the mass of sodium—correspond to the sodium adducts of oligo(*δ*-valerolactone) chains terminated by benzyl ester (derived from the initiator) and hydroxyl end groups. The next two series of ions observed on mass spectrum and visible on each step of oligomerization in mass spectrum (labeled as B and B’ series) that appear at *m/z* = 17 + (n × 100) + 1 + 23 and *m/z* = 17 + (n × 100) + 23 + 23 correspond to the adducts of oligo(*δ*-valerolactone) chains terminated by carboxyl and hydroxyl end groups (B series) on one hand and the same oligomer chains but in the form of carboxylic sodium salt (series B’) on the other hand. 

Additionally, the series of ions with lower relative intensity labeled as series C (see Figure 3, that occurs at *m/z* = (n × 100) + 23 was observed in the mass spectrum. This series represents cyclic PVL oligomers. These cyclic oligomers (mostly visible in the lower mass range) can be produced by backbiting degradation of linear polymer [34]. The general structures of the ions present in the ESI-MS spectrum (Figure 2) are shown in Figure 3. 

**Figure 4:**
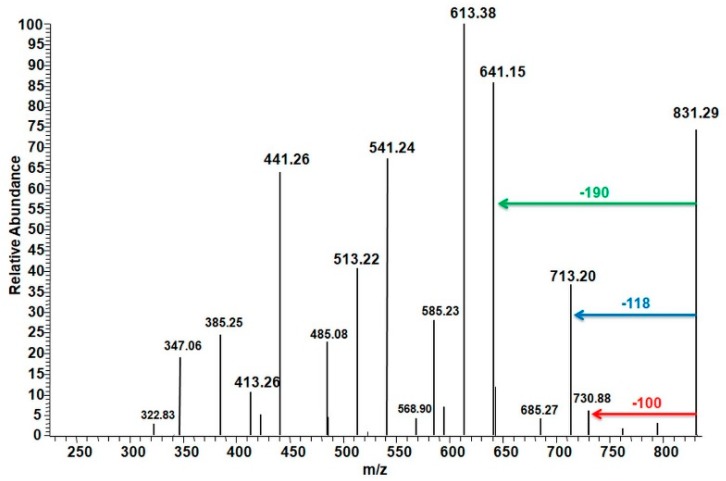
ESI-MS/MS spectrum of the sodium adducts of poly(*δ*-valerolactone) oligomers with benzyl and carboxyl end groups at *m/z* 831 (Sample 1, Table 1).

Further information about the structure of polymer chains was obtained by ESI tandem mass spectrometry studies, where the parent ions of interest were separated from all other peaks based on their *m*/*z* values and was further fragmented using the CID technique. The fragmentation pattern was used to diagnose the chemical structure of polymer end groups. To verify the structural assignment of the individual PVL macromolecules, the ESI-MS/MS fragmentation experiments for the ions selected from the main series A and B were performed. The ESI-MS/MS spectrum of the sodium adduct of PVL oligomers with benzyl and hydroxyl end groups at *m/z* 831 belonging to series A is presented in Figure 4. The fragmentation of this PVL oligomers at *m/z* 831 can take place by losses of neutral molecules of 5-hydroxyvaleric acid (118 Da) and benzyl ester of 4-pentenoic acid (190 Da) from the ends of PVL oligomers as well as a result of random breaking of the ester bonds along the oligomers chain (β-hydrogen rearrangements); Specifically, the ion at *m/z* 713 is formed due to the loss of neutral (5-hydroxyvaleric acid), and the ion at *m/z* 641 is formed due to the loss of benzyl ester of 4-pentenoic acid (190 Da), which confirms the oligomers beginning to fragment from the benzyl end and the hydroxyl end group via the breakage of ester bonds along the polyester chain. It can also be concluded that the attack of hydroxyl end groups on neighboring carbonyl carbon and subsequent loss of neutral molecule of cyclic *δ*-valerolactone (100 Da) may allow the start of fragmentation of the benzyl- and hydroxyl-terminated parent ion. Thus, the fragment ion at *m/z* 731 may be formed by the release of neutral molecule of cyclic *δ*-valerolactone (100 Da) of 4-pentenoic acid.

**Scheme 2:**
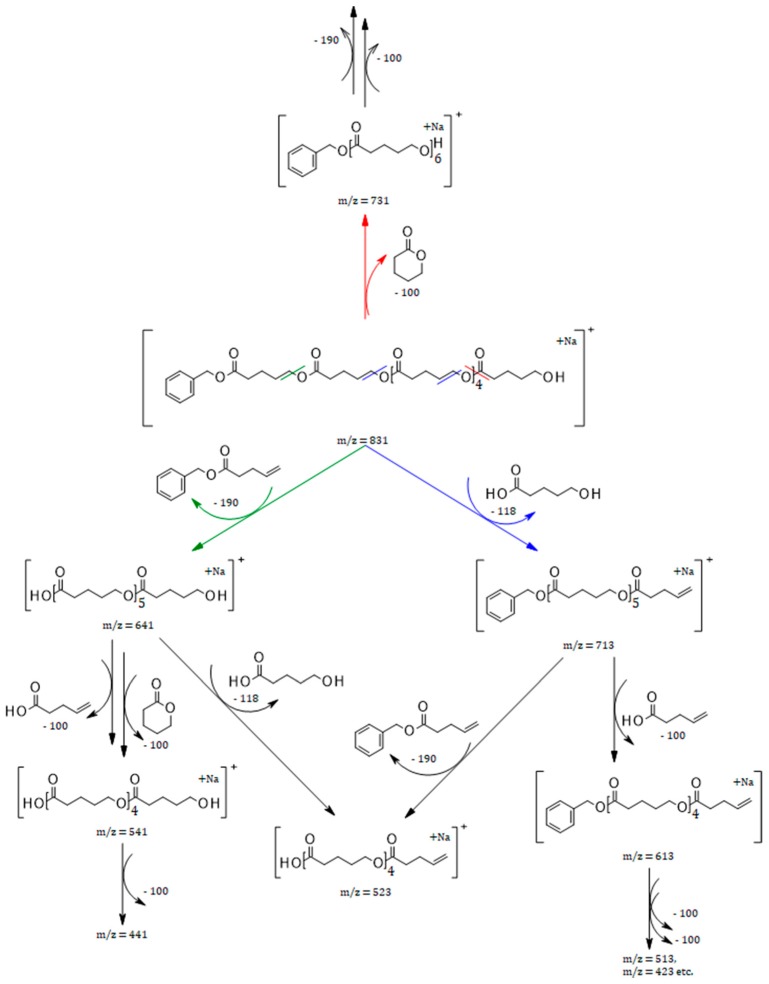
Possible fragmentation pathways for sodium adducts of benzyl- and hydroxy-terminated PVL oligomers at *m/z* 831.

Based on our knowledge about the fragmentation mechanism of different polyester chains [31,34], the possible fragmentation pathways for sodium adduct of benzyl- and hydroxyl-terminated PVL oligomer at *m/z* 831 is presented in Scheme 2.

**Figure 5:**
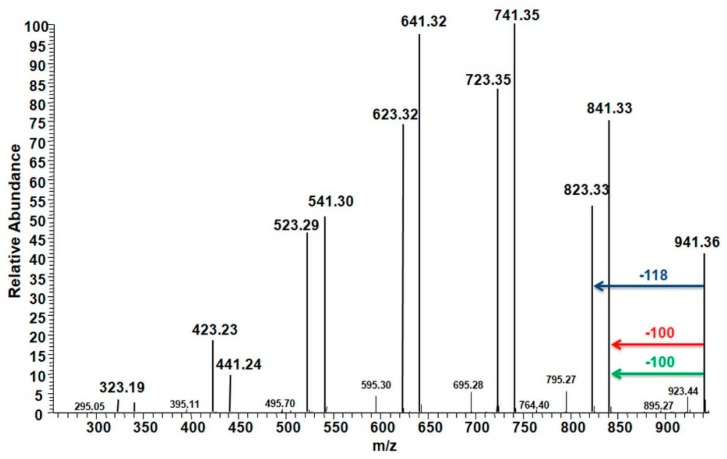
ESI-MS/MS spectrum of the sodium adducts poly(*δ*-valerolactone) oligomers with hydroxyl and carboxyl end groups at *m/z* 941 (Sample 1, Table 1).

**Scheme 3:**
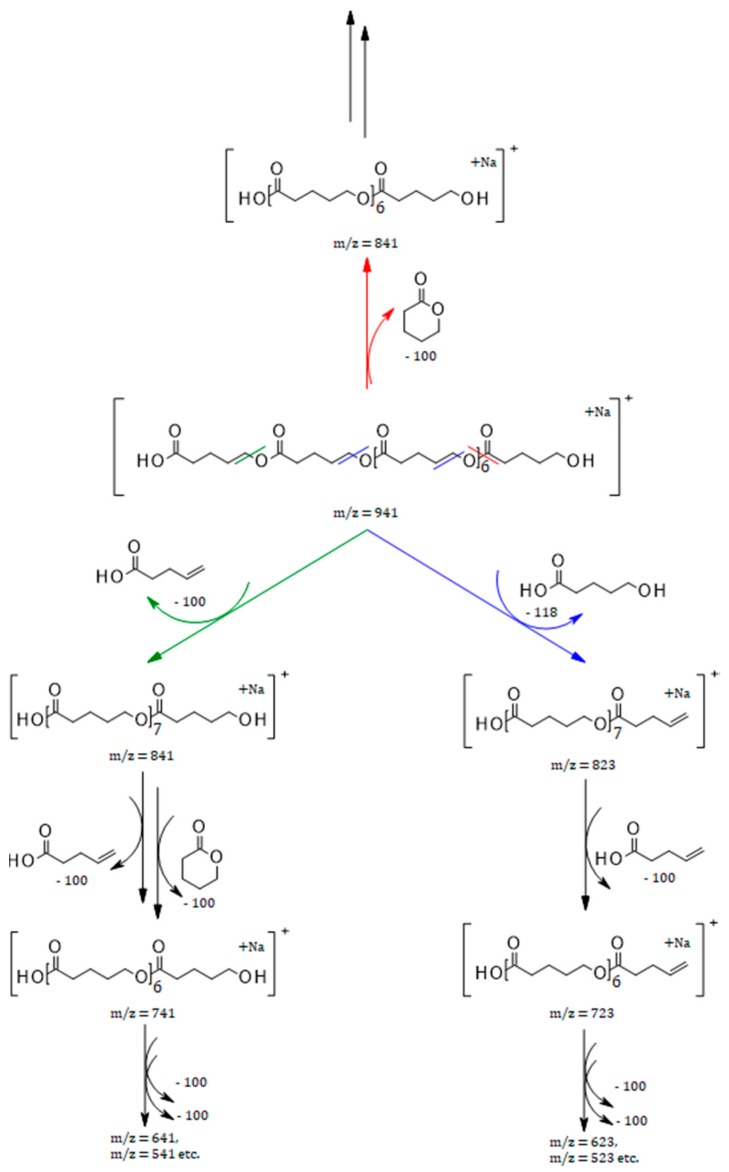
Possible fragmentation pathways for sodium adducts of benzyl- and hydroxy-terminated PVL oligomers at *m/z* 941.

Figure 5 shows the ESI-MS/MS spectrum of the sodium adduct of PVL oligomers terminated by hydroxyl and carboxyl end groups at *m/z* 941 and belonging to the series B. The fragmentation of this oligomer parent ions—which can take place as a result of random breaking of the ester bonds along the oligomer chain as well as losses of neutral molecule of *δ*-valerolactone (100 Da)—leads to the formation of two series of fragment ions. The first series of fragment ion at *m/z* 841, 741, 641, 541, and 441 can be formed by the successive loss of the neutral molecules of *δ*-valerolactone (100 Da) or 4-pentenoic acid, respectively. The second series of fragment ions at *m/z* 823, 723, 623, 523, 423, and 323 correspond to the oligomers formed due to the loss in first step of a neutral molecule of 5-hydroxypentanoic acid 5-hydroxyvaleric acid (118 Da) and then via the random breakage of ester bonds along the polyester chain and/or successive losses of 4-pentenoic acid. Based on our knowledge about the fragmentation mechanism of different polyester chains [31], the possible fragmentation pathway of the sodium adduct of PVL oligomers at *m/z* 941 was proposed; this is presented in Scheme 3.

A comparison of the fragmentation spectra presented in Figure 4 and Figure 5 with the respective theoretical fragmentation pathways shown in Scheme 2 and Scheme 3 clearly indicated that the main A series corresponded to sodium adducts of PVL chains terminated by benzyl ester (derived from the initiator) and hydroxyl end groups (Figure 4, Scheme 2), while series B was ascribed to PVL oligomers with hydroxyl and carboxyl end groups (Figure 5, Scheme 3).

The structure of the PVL was additionally confirmed using the triple quadrupole (QqQ) instrument by fragmentation of lithium adducts of PVL oligomers terminated by benzyl ester and hydroxyl end groups and with carboxyl and hydroxyl end groups. The details concerning the ESI-MS/MS analysis of Sample PVL1 using QqQ instrument and the resulting mass spectra are represented in Figures 1S–3S in Supplementary Material.

### 2.3. MALDI TOF-MS 

MALDI TOF-MS was used to complete the structural characterization of oligomer mixture that are present in the samples of PVL oligomers synthesized and to check if composition of those oligomers depended on the average molar mass of the poly(*δ*-valerolactone) obtained.

**Figure 6:**
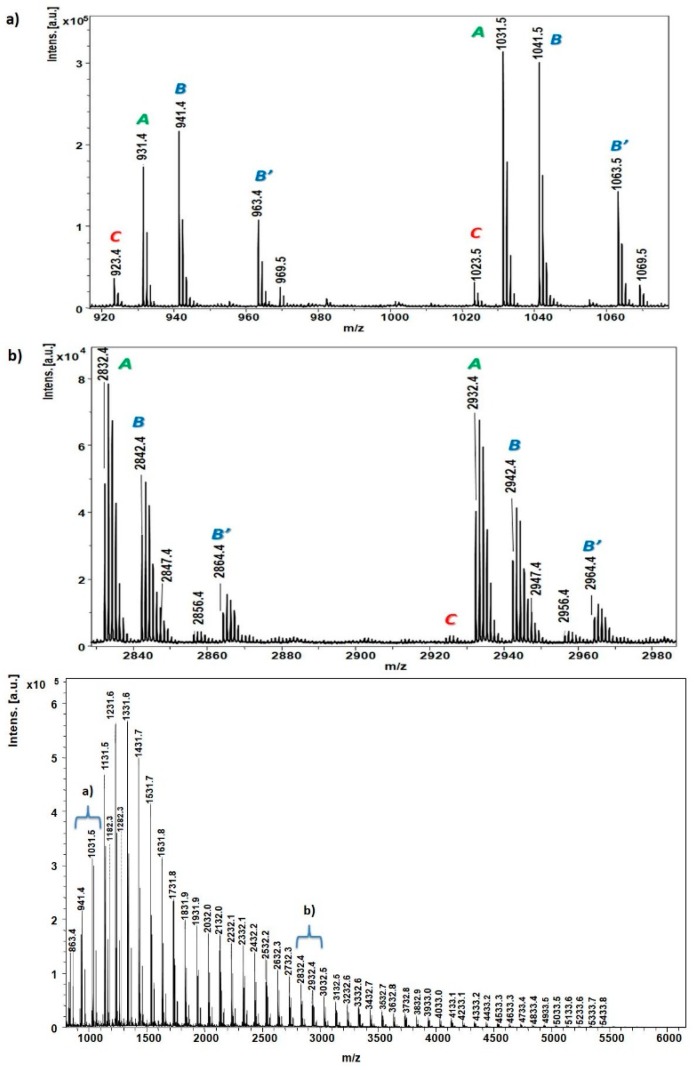
MALDI-TOF-MS spectrum (positive ion mode) of poly(*δ*-valerolactone) together with two spectral expansions in the mass range: (**a**) *m/z* 910–1100 and (**b**) *m/z* 2810–3090 (Sample 2, Table 1).

Figure 6 shows MALDI-TOF mass spectrum (positive ion mode) of poly(*δ*-valerolactone) with *M_n_* of 4500 g/mol and *M_w_/M_n_* 1.5, together with two spectral expansions in the mass range *m/z* 910–1100 and *m/z* 2810–3090.

In Figure 6, it can be observed that the PVL species are indeed disperse, with two main regions being identified—a high intensity region between *m/z* 800 and *m/z* 2000 and a lower intensity region from *m/z* 2000 up to *m/z* 6000. However, from the mass spectrum obtained of a sample with a higher average molar mass, it could be seen that regardless of the mass range of the sample (see spectral expansions Figure 6a,b) on each stage of oligomerization, the four series of ions were visible. Based on the *m/z* value of the signals present in MALDI mass spectrum and to ESI-MS analysis, two main series of ions could be identified—series A, which corresponded to sodium adducts of benzyl alcohol initiated PVL chains and series B, which represented sodium adducts of PVL chains with OH and COOH end groups (see Figure 3). Two minor series B’ and C were also present and corresponded to sodium adduct of PVL linear chains with OH and COONa end groups and to sodiated cyclic PVL oligomers, respectively. The structures of the ions present in MALDI mass spectrum were identical to the one in ESI-MS (see Figure 3). The only difference related to relative intensities of cyclic oligomers (series C), which tended to decrease at higher *m/z* values as observed in the enlarged view of the MALDI spectrum (Figure 6b) around *m/z* 3000. The formation of cyclic oligomers via backbiting reaction indicated that intramolecular transesterification reactions took place. 

### 2.4. ESI-MS Negative Ion Analysis 

**Figure 7:**
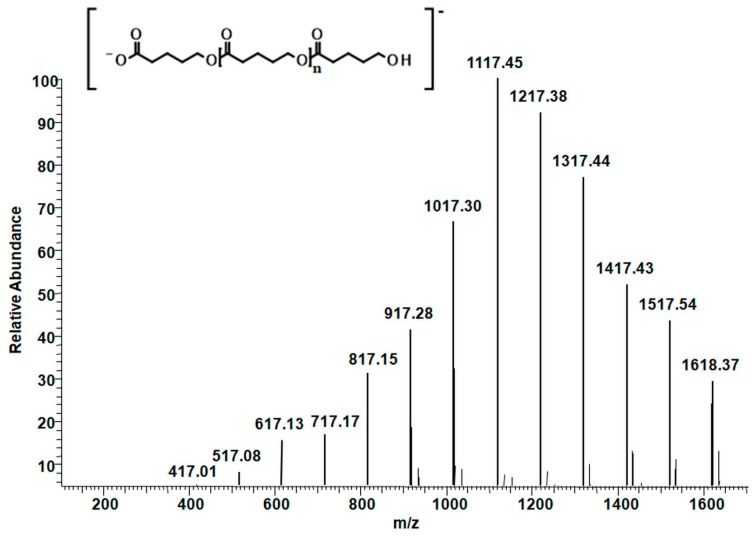
ESI-MS^n^ spectrum (negative ion mode) of poly(*δ*-valerolactone) precursor ion at *m/z* 917 and the respective parent ions with different chain length (MS^2^–MS^9^), (Sample 1, Table 1).

Figure 7 shows the ESI-MS (negative ion mode) of PVL oligomers. This spectrum clearly highlights only one main series of singly charged ions with a peak-to-peak mass increment of 100 Da. Based on *m/z* value of ions that appears on this spectrum at *m/z* = 17 + (n × 100), the observed series of ions can be assigned to PVL oligomers terminated by hydroxyl and carboxylic end groups. 

Only this type of oligomers among the oligomers present in the synthesized PVL samples can be ionized under the conditions of ESI-MS analysis in negative ion mode and created singly charged anions.

**Scheme 4:**
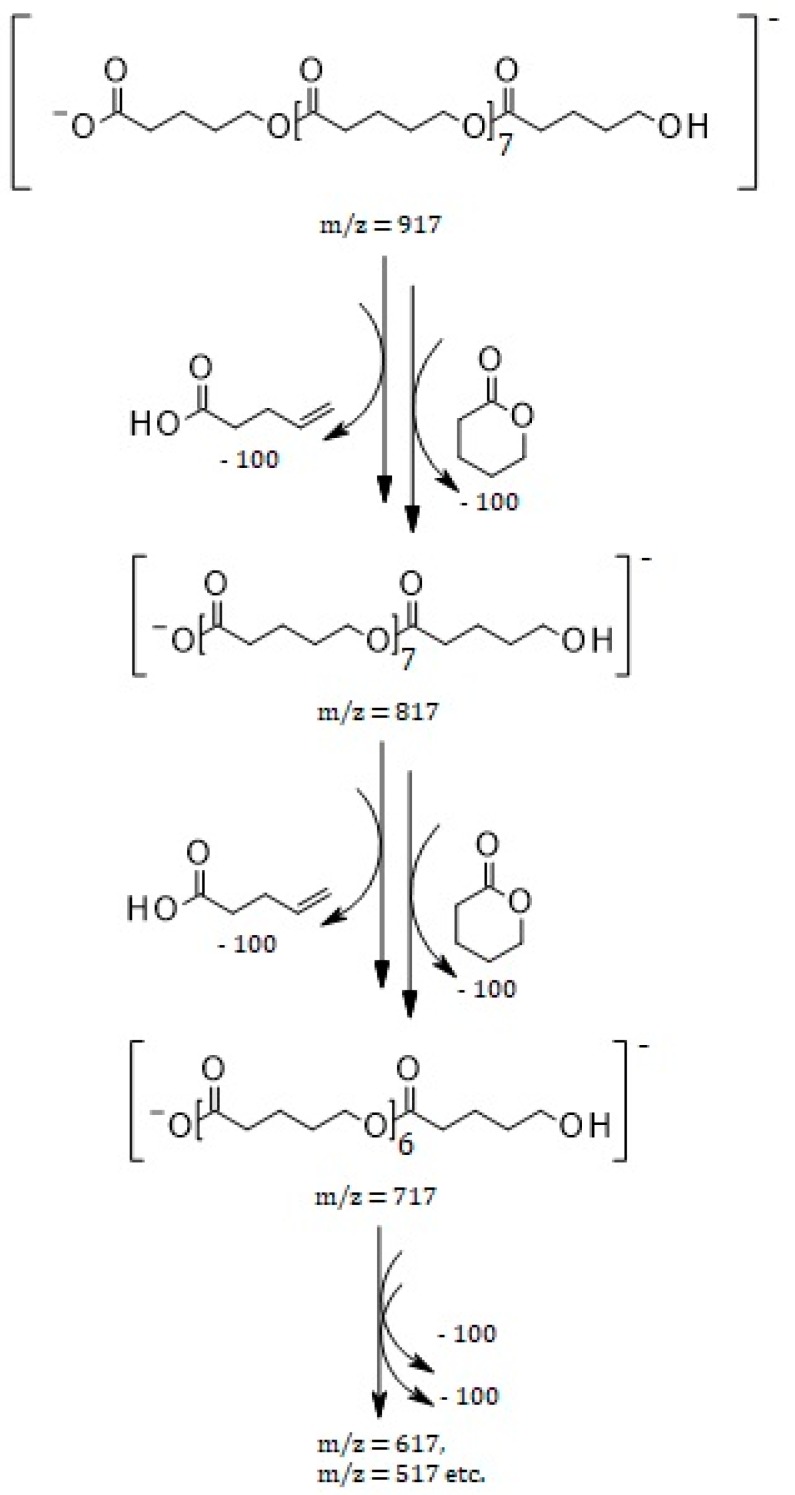
Possible fragmentation pathways of the anion of PVL oligomers terminated by hydroxyl and carboxylic end group.

**Figure 8:**
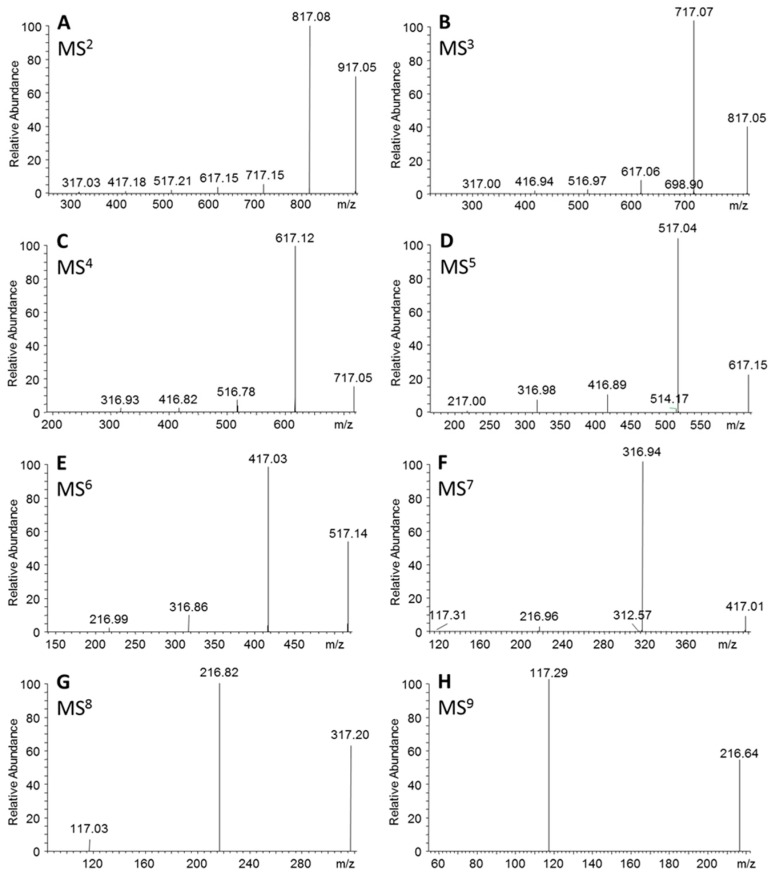
ESI-MS^n^ spectra (negative ion mode) of poly(*δ*-valerolactone) precursor ion at *m/z* 917 and the respective parent ions with different chain length (MS^2^ (A)–MS^9^ (H)), (Sample 1, Table 1).

Figure 8 shows the ESI-MS^n^ (negative ion-mode) fragmentation spectra obtained for the PVL oligomer with nine repeating units at *m/z* 917. Interestingly, in all MS^n^ fragmentation experiments (from MS^2^ up to MS^9^), it can be clearly seen that the fragmentation of the ion at *m/z* 917 leads to creation stable species and only one set of fragment ions is observed. The possible fragmentation pathway of the anion of PVL oligomers terminated by hydroxyl and carboxylic end groups is presented in Scheme 4. 

However, from the inspection of the fragmentation spectra (from MS^2^ to MS^9^) of this ion (Figure 8), it can be concluded that it is more likely fragmentation of this parent anion occurs from hydroxyl end. The attack of hydroxyl end groups on neighboring carbonyl carbon and subsequent loss of neutral molecule of cyclic *δ*-valerolactone (100 Da) allows the start of fragmentation of these ions. This hypothesis is confirmed by the high intensity of the first fragmentation ion present in MS^2^-MS^9^ spectra as well as the presence in the MS^7^-MS^9^ experiment of the anion of 5-hydroxyvaleric acid (117 Da).

## 3. Materials and Methods 

### 3.1. Gel Permeation Chromatography (GPC)

Molecular weight and mass distribution measurements were performed by GPC. The experiments were carried out in CHCl_3_, (GPC Solvent, stabilized with amylene, purity 99.8%, (Fischer Chemical, Loughborough, UK) with a flow rate of 0.35 mL/min at 30 °C using a Spectra-Physics 8800 solvent delivery system with two Mixed C PL-gel 5 µm MIXED-C ultra-high efficiency columns (Agilent, Santa Clara, CA, USA) in series with a mixed bed and a linear range of M_w_ 200–2,000,000 and a Shodex SE 61 refractive index detector (Showa Denko, Munich, Germany). A 10 μL of sample solutions in CHCl_3_ (concentration 0.5% *w/v*) were injected into the system. Polystyrene standards (calibration Kit S-M-10, Polymer Laboratories) with low polydispersity were used to generate a calibration curve. The samples were measured using OmneiSEC 4.1 (Vescotek) software (Malvern, Worcestershire, UK) 

### 3.2. Proton Nuclear Magnetic Resonance (^1^HNMR)

^1^H-NMR spectra were acquired using a Bruker-Advance apparatus (Bruker BioSpin GmbH, Rheinstetten, Germany) operating at 600 MHz with Bruker TOPSPIN 2.0 software using tetramethylsilane (TMS) as an internal standard in deuterated chloroform (CDCl_3_) at 25 °C. Spectra were recorded with 64 scans, an 11 μs pulse width, and a 2.66 s acquisition time.

### 3.3. Electrospray Ionization Mass Spectrometry (ESI-MS^n^) Analysis

ESI-MS^n^ analysis was performed using a Thermo LCQ Fleet ion trap mass spectrometer (Thermo Fisher Scientific Inc., San Jose, CA, USA). The samples studied were dissolved in chloroform/methanol (1:1 *v/v*), and the solutions were introduced to the ESI source by continuous infusion using the instrument syringe pump at a rate of 10 µL/min. The ESI source of the LCQ was operating at 5.0 kV, and the capillary heater was set to 200 °C. Nitrogen was used as the nebulising gas. 

For ESI-MS/MS^n^ experiments, monoisotopic ions of interest were isolated in the ion trap and activated using helium damping gas in the mass analyzer to promote collisions (CID). The RF amplitude was set such that the peak height of the molecular ion decreased by at least 50%. In addition, the ESI-MS/MS experiments were performed using a Micromass/Waters QqQ Quattro II mass spectrometer equipped with a Z-spray ionization source. The analyses were performed in the positive and negative ion modes.

### 3.4. Matrix Assisted Laser Desorption Ionization (MALDI-TOF-MS) 

Polymer samples after evaporation of solvent were dissolved in THF to a concentration of 10 mg/mL. Samples were mixed with a matrix solution 1,8,9-antracenetriol or dithranol (Sigma-Aldrich, Merck, Darmstadt, Germany). (10 mg/mL in a ratio of 1/100 (*v/v*). 1 μL of this mixture were deposited on polished steel MALDI target (Bruker, Bremen, Germany). Mass spectra of polymers were measured on UltrafleXtreme TOF instrument (Bruker, Bremen, Germany), equipped with a 355 nm smartbeam-2 laser capable of pulsing frequency 1 kHz. Mass spectrometer was operated by FlexControl 3.3 software (Bruker, Bremen, Germany). Acquired spectra were processed by FlexAnalysis 3.3 software (Bruker, Bremen, Germany). The ionization laser power was adjusted just above the threshold in order to produce charged species. The mass spectra were collected in amount of above 10,000 spectra. The fragmentation spectra of the selected MS peaks were acquired in LIFT mode, setting the laser power just above the threshold to obtain parent ions fragmentation.

### 3.5. Homopolymerization of δ-Valerolactone

*δ*-Valerolactone (*δ*-VL, technical grade Sigma-Aldrich, Merck, Darmstadt, Germany) was freshly distilled from CaH_2_. Benzyl alcohol (BnOH, 99.80%, (Sigma-Aldrich, Merck, Darmstadt, Germany.) and boric acid (B(OH)_3_, 99.5%,( Sigma-Aldrich, Merck, Darmstadt, Germany) were used as received. The polymerization of *δ*-valerolactone was conducted according to the method of Ren et al. [7], with some modification. The sample of *δ*-valerolactone (5.56 g, 48 mmol, 50 equiv.) was placed in a flame-dried 50 mL two-necked round-bottom flask under nitrogen atmosphere. Benzyl alcohol (0.1 mL, 0.960 mmol, 1.0 equiv.) was added, and the flask was placed in a thermostated oil bath at 130 °C. Boric acid (0.065 g, 0.960 mmol, 1.0 equiv.) was added with vigorous stirring under a nitrogen atmosphere to start the polymerization. The reaction was quenched after 70 h by the addition of Amberlyst 24 (Sigma-Aldrich, Merck, Darmstadt, Germany.) ion exchange resin and dissolving in dichloromethane and by the addition of a large excess of cold methanol. After separation of the solvent and any unreacted monomer from the precipitated products, the polymers were dried under vacuum prior to characterization. ^1^H-NMR (600 MHz, CDCl_3_, ppm): 1.24 (m CH_2_(CH_2_)CH_2_), 1.67 (m -COCH_2_CH_2_CH_2_CH_2_O-), 2.33 (t, -COCH_2_(CH_2_)_3_O-), 3.64 (t, CO(CH_2_)_3_CH_2_OH), 4.07 (t, -CO(CH_2_)_3_CH_2_O), 4.32 (2H,-), 5.10 (t, PhCH_2_O end group), 7.34 (5H, aromatic).

## 4. Conclusions

Biodegradable PVL oligomers were obtained via a bulk-ring opening polymerization of δ-valerolactone and boric acid as a catalyst. The boric acid catalyst eliminates the need for the use of a toxic organic or metal-based catalyst, which may in turn increase the suitability of these polymer materials for use in biomedical purposes.

The molecular structure of the obtained PVL oligomers was determined with the aid of ESI-MS and MALDI-TOF mass spectrometry supported by NMR analyses. It was shown that the PVL samples obtained were a mixture of three types of PVL oligomers differing in degree of oligomerization and chemical structures of the end groups. The presence of two main types of linear PVL polyester chains—one terminated by benzyl ester and hydroxyl end groups and one with carboxyl and hydroxyl end groups—were confirmed with application of tandem ESI-MS/MS experiments. Additionally, a small amount of cyclic PVL oligomers with low oligomerization degree was identified in the studied samples.

Thus, the presented results demonstrate the utility of MS techniques for the analysis of individual molecules of biodegradable PVL oligoesters studied. These results are important, and the presented MS methods and protocols can be used for the structural characterization at the molecular level of designed biodegradable polyester materials with potential medical applications.

## Figures and Tables

**Table 1:** The number average molecular weight (*M_n_*) and dispersity (*M_w_/M_n_*) of the poly(*δ*-valerolactone) samples characteristics by GPC.

Sample	*M_n_* (g/mol^−1^)	*M_w_/M_n_*
PVL1	1500	1.5
PVL2	4500	1.4

## Supplementary Materials

The following are available online.
