# Supplementary material for: Molecular Level Structure of Biodegradable Poly(Delta-Valerolactone) Obtained in the Presence of Boric Acid
Source: Molecules. 2018 Aug 14;23(8):2034. doi: 10.3390/molecules23082034 (PMC6222617; doi:10.3390/molecules23082034)
Supplement: Supplementary file 1 [file molecules-23-02034-s001.zip › molecules-342208-SI.pdf]

SUPPLEMENTARY MATERIAL

Molecular level structure of biodegradable poly(delta-valerolactone) obtained in the presence of boric acid

Khadar Duale[1*], Magdalena Zięba [1], Paweł Chaber [1], Dany Jeanne Di Fouque[2], Antony Memboeuf [2], Cristian Peptu[3,4], Iza Radecka[5], Marek Kowalczuk[1,5] and Grażyna Adamus[1*]

1 Centre of Polymer and Carbon Materials, Polish Academy of Sciences, 34, M. Curie-Sklodowska St., 41-819 Zabrze, Poland; email: grazyna.adamus@cmpw-pan.edu.pl;

2 CEMCA, Université de Bretagne Occidentale, 6 Av. Le Gorgeu, 29238 Brest Cedex 3, France; memboeuf@univ-brest.fr

3 Polymer Institute, Slovak Academy of Sciences, Dúbravská cesta 9, 845 41 Bratislava 45, Slovakia; upolcris@savba.sk;

3 "Petru Poni" Institute of Macromolecular Chemistry, Alee Grigore Gica Voda 41A, 700487 Iasi, Romania; cristian.peptu@icmpp.ro

5 School of Biology, Chemistry and Forensic Science, Faculty of Science and Engineering, University of Wolverhampton, Wolverhampton WV1 1SB, UK; M.Kowalczuk@wlv.ac.uk

* Correspondence: grazyna.adamus@cmpw-pan.edu.pl; kduale@cmpw-pan.edu.pl; Tel.: + 48 32 2716077

The ESI-MS/MS analysis of Sample PVL1 using QqQ instrument

The ESI tandem mass spectrometry (ESI-MS/MS) experiments were performed using a Micromass/Waters QqQ Quattro II mass spectrometer equipped with a Z-spray ionization source. Both positive and negative ion modes were used to analyze the sample. Polymer samples were dissolved at 5.10-4 M in a mixture of water/acetonitrile/methanol (1/1/1). Acetonitrile and methanol, at HPLC-MS grade, were purchased from Sigma-Aldrich (Illkirchen, Germany) and, de-ionized ultra-pure water (18 MΩ.cm resistivity) was obtained using Milli-Q Integral 3 Water System (Merck Millipore, Guyancourt, France). In the positive ion mode, LiCl was added to reach a final concentration of 10-3 M. The solutions were introduced by direct injection and electrosprayed via a syringe pump at 60 µL/min flow rate. The temperature of the source was set at 80 °C, nitrogen was used as desolvation gas at a flow rate of 350 L/h and a temperature of 160 °C. The electrospray voltages were set with the following main parameters: capillary at 3.0 kV, sample cone at 80 V and extraction cone at 10 V. The first hexapole was set in RF-mode only for an improved ion transmission. The first and the second quadrupole analysers were used with Low-Mass (LM) and High-Mass (HM) resolutions set to 17, or to 10 for the first quadrupole during the isolation stage in MS/MS experiments. Argon was used as a collision gas with a pressure of 4.10-3 mbar in the collision cell during MS/MS experiments, while vacuum was maintained at about 4.10-5 mbar inside the instrument (uncorrected gauge reading). The MassLynx software version 4.0 (Waters, Manchester, UK) was used for data acquisition and mass spectra processing.

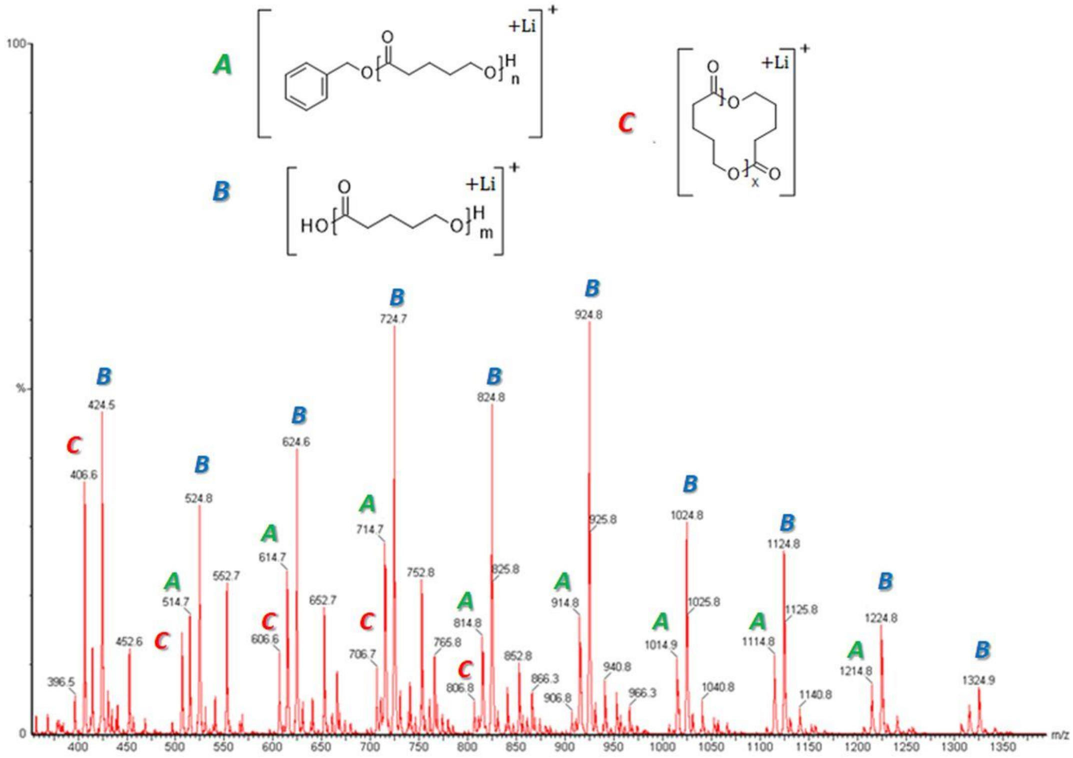

Figure 1S. MS spectrum obtained using ESI-QqQ instrument (in positive ion mode) showing the hydroxyl end-groups and mono-substituted benzyl end-groups of PVL1 sample.

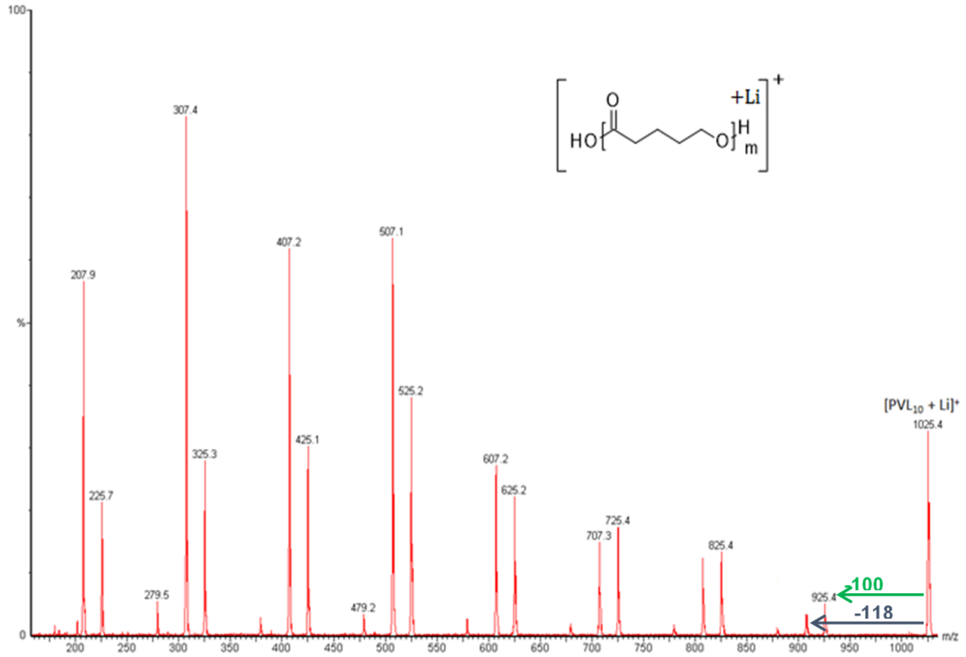

Figure 2S. MS/MS spectrum for lithiated cationized H-PVL10-OH (*m/z* 1025) acquired on ESI-QqQ instrument.

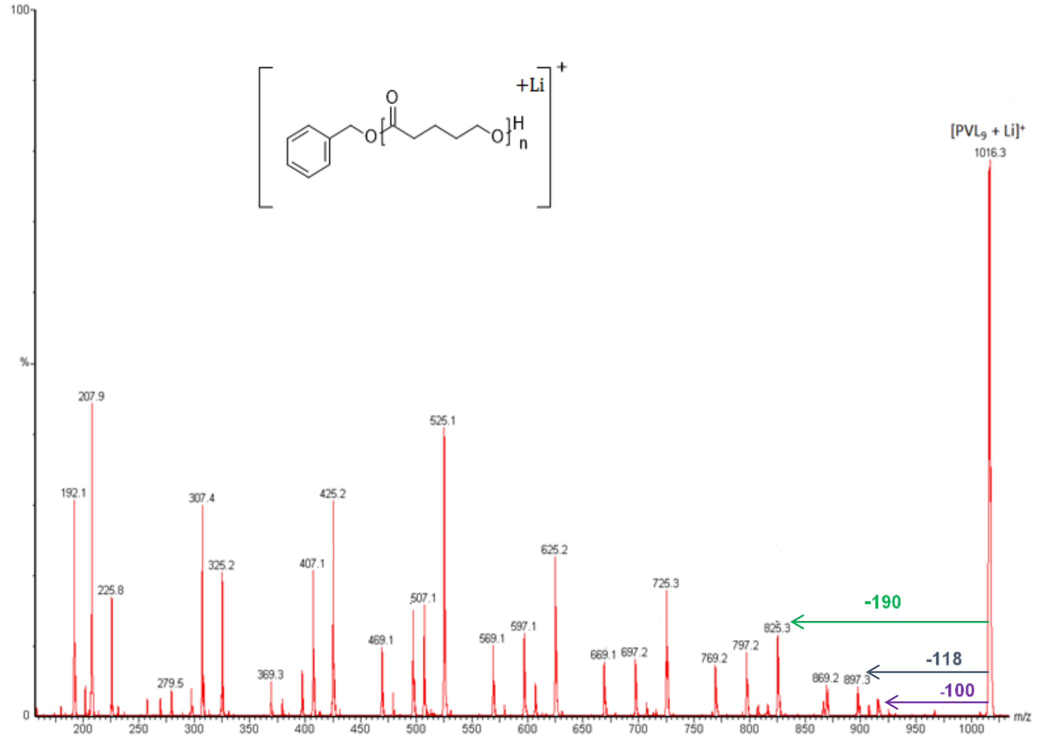

Figure 3S. MS/MS spectrum for lithium cationized H-PVL9-O-benzyl (*m/z* 1016) acquired on ESI-QqQ instrument.
